# Immunohistochemical localization of CD1a-positive putative dendritic cells in human breast tumours

**DOI:** 10.1038/sj.bjc.6690150

**Published:** 1999-02

**Authors:** E E Hillenbrand, A M Neville, B J Coventry

**Affiliations:** Department of Surgery, University of Adelaide, Royal Adelaide Hospital, Adelaide, SA 5000, Australia

**Keywords:** CD1a, dendritic cells, immunohistochemistry, breast, tumour, human, cancer

## Abstract

The presence of a high number of infiltrating CD1a^+^cells in malignant neoplasms has been reported to be associated with an improved prognosis, reduced tumour recurrence and fewer metastases. This study identified a population of CD1a^+^cells within the lymphoid cell infiltrate in human ductal breast carcinoma (*n*= 52), which was significantly different from normal breast tissue, in which only two out of nine cases expressed CD1a^+^cells (*P*= 0.0192). In the majority of cases, the infiltrate was low compared with the number of macrophages and T cells present (results not shown). There was no correlation between the number of CD1a^+^cells and tumour grade, with all tumour grades expressing similar numbers of infiltrating CD1a^+^cells. There was clear evidence, however, that the CD1a^+^cells were closely associated with tumour cells. It is likely that CD1a^+^cells have a role in antigen capture and presentation in human tumours, and this study documents the density of CD1a^+^cells in a large sample of all histological grades of human breast carcinomas. *1999 Cancer Research Campaign*


*British Journal of Cancer* (1999) **79**(5/6), 940–944
© 1999 Cancer Research Campaign
Article no. bjoc.1998.0150

### Immunohistochemical localization of CD1a-positive putative dendritic cells in human breast tumours

**EE Hillenbrand, AM Neville and BJ Coventry**

Department of Surgery, University of Adelaide, Royal Adelaide Hospital, North Terrace, Adelaide SA, 5000, Australia

**Summary** The presence of a high number of infiltrating $CD1a^+$ cells in malignant neoplasms has been reported to be associated with an improved prognosis, reduced tumour recurrence and fewer metastases. This study identified a population of $CD1a^+$ cells within the lymphoid cell infiltrate in human ductal breast carcinoma ($n = 52$), which was significantly different from normal breast tissue, in which only two out of nine cases expressed $CD1a^+$ cells ($P = 0.0192$). In the majority of cases, the infiltrate was low compared with the number of macrophages and T cells present (results not shown). There was no correlation between the number of $CD1a^+$ cells and tumour grade, with all tumour grades expressing similar numbers of infiltrating $CD1a^+$ cells. There was clear evidence, however, that the $CD1a^+$ cells were closely associated with tumour cells. It is likely that $CD1a^+$ cells have a role in antigen capture and presentation in human tumours, and this study documents the density of $CD1a^+$ cells in a large sample of all histological grades of human breast carcinomas.

**Keywords:** CD1a; dendritic cells; immunohistochemistry; breast; tumour; human; cancer

The phenotype of tumour-infiltrating lymphocytes (TILs) in solid tumours, including breast tumours, has been well documented using monoclonal and polyclonal antibodies on frozen and paraffin tissue sections (Feradini et al, 1993; Head et al, 1993). Dendritic cells (DCs) are potent antigen-presenting cells (APCs), whose role is to capture and process antigen via the MHC II pathway for presentation to $CD4^+$ T cells. This results in T cell-mediated immune responses against cells carrying specifically recognizable antigenic targets (Williams et al, 1994). The presence of DCs in human tumours has been associated with a better prognosis, reduced tumour recurrence and fewer metastases (Huang et al, 1990; Becker, 1992; Tsujitani et al, 1992; Inoue et al, 1993). DCs have therefore been proposed as playing a central role in the generation of an effective anti-tumour immune response (Becker, 1992).

The CD1a molecule is classed as a ‘non-conventional’ antigen-presenting molecule with significant homology to ‘conventional’ MHC class I and MHC class II molecules (Hanau et al, 1994) and is integral in peptide acquisition and antigen presentation (Porcelli et al, 1989). Many tumours of different histologies contain populations of CD1a-positive putative DCs within the lymphoid cell infiltrate (Becker, 1992). Studies of gastric carcinoma (Huang et al, 1990; Tsujitani et al, 1992) and transitional cell carcinoma (Inoue et al, 1993) have shown an inverse correlation between tumour grade and DC infiltrate. However, the distribution and density of DCs within infiltrating ductal carcinoma (IDC) and ductal carcinoma in situ (DCIS) of the breast has not been previously described.

Within the normal lympho reticular system, only Langerhans cells (LCs), some skin DCs (Yu et al, 1989; Osterhoff et al, 1994; Williams et al, 1994) and some thymic cells (Williams et al, 1994) express CD1a. No other normal lymphoid cells have been shown to express CD1a. High CD1a and MHC molecule expression on LCs/DCs may explain their potency as APCs. The expression of CD1a appears to be dependent on the functional state of the DCs, as CD1a expression is high when cells are capturing antigen and is down-regulated when antigen presentation is occurring (Moulon et al, 1991). Freshly isolated bone marrow and blood DCs are CD1a negative, as are most APCs resident in lymphoid organs (Williams et al, 1994). In addition to CD1a, the polyclonal antibody S100 has been used by several groups as a DC identification marker (Nakano et al, 1989; Huang et al, 1990; Tsujitani et al, 1992; Inoue et al, 1993). This antibody reacts with the cytoplasmic protein S-100b, which is present in neurones, melanocytes and some DCs (Krenacs et al, 1993).

It is unclear why the anti-tumour immune response is ineffective; however, possible mechanisms include low DC numbers, poor antigen capture or presentation, failure of DC maturation, lack of IL-2 cytokine production, down-regulation of cytokine receptors (Coventry et al, 1996) and failure of expression of co-stimulatory molecules such as B7-1 or B7-2 (June et al, 1994) or CTLA4 ligand (Johnson and Jenkins, 1992). The number of DCs in human breast carcinomas has not been sufficiently documented, therefore this study aimed to identify putative DCs in breast tumours. Anti-CD1a monoclonal antibody (MAb) was used on fresh-frozen tissue sections to (1) investigate DC density, (2) examine the spatial relationship of DCs to tumour and immunological cells and (3) correlate the DC infiltrate with different histological tumour grades (Scarfe/Bloom and Richardson grading system).

*Received 2 January 1998*
*Revised 3 July 1998*
*Accepted 13 July 1998*

*Correspondence to:* BJ Coventry Department of Surgery, University of Adelaide, Royal Adelaide Hospital, North Terrace, Adelaide, SA, 5000, Australia

### METHODS

### Tissues and processing

Frozen tissue was obtained from 52 ductal breast carcinomas (by tumour grade: DCIS, 7; IDC I, 11; IDC II, 19; IDC III, 15), with nine non-tumour control samples obtained from breast tissue distant from the tumour site. Control samples were histologically free of tumour cells. Tissue was embedded in OCT (Tissue Tek, Miles Laboratories) and snap frozen in liquid nitrogen. Serial sections (4 μm) were cut (Ames Cryostat, Tissue Tek) onto gelatinized slides, acetone fixed (10 min) and air dried.

### Immunohistochemical techniques

Antibodies were mouse anti-human monoclonals with the exception of S-100 (rabbit anti-human polyclonal) diluted in 10% normal horse serum (NHS, Sigma) at varying concentrations: CD1a at 1:1000 (Dako, Denmark), S100 at 1:80 000 (Dako, Denmark), Cam 5.2 at 1:500 (Becton Dickinson), isotype controls IgG1, IgG2a (supernatants at 1/10, L. Ashman, IMVS Adelaide), second antibody biotinylated rabbit anti-mouse IgG/IgM at 1/500 (Dako) and streptavidin–HRP at 1:1000 (Pierce, USA).

#### *Single immunostaining protocol*

All reactions were carried out at room temperature with phosphate-buffered saline (PBS) washes in triplicate between incubations. Sections were blocked with 10% NHS (30 min), incubated overnight in a humidified chamber with the primary antibody, followed by biotinylated second antibody incubation (1 h). Endogenous peroxidase blockade (0.5% hydrogen peroxide; methanol, 20 min) was followed by streptavidin–HRP incubation (1 h). High-sensitivity nickel chloride-enhanced diaminobenzidene (nickel DAB) techniques were used (Coventry et al, 1994, 1995), and sections were counterstained with methyl green.

#### *Double staining protocol*

The primary antibody concentrations were increased, and incubation times were shortened to 1 h. The first primary antibody was visualized using nickel DAB, and the second primary antibody was visualized using DAB/imidazole.

### Identification of putative DCs

There was no single specific MAb that identified DCs. Two of the most commonly used MAbs, anti-CD1a and anti-S100, were tested and compared to assess the MAb that produced the most reliable and consistent staining results in breast tumour tissue. The sections were visually assessed for (1) the presence or absence of specific cell staining that demonstrated distinctive DC morphology and (2) comparison of the location of positive cells by relationship to tissue landmarks in serial sections using different MAbs.

### Identification of tumour cells

Epithelial cells within the tumour were stained with Cam 5.2, a MAb that reacts with secretory epithelia of normal tissue as well as epithelial adenocarcinomas (Makin et al, 1984).

### Characterization of cellular infiltrate

Infiltration of $CD1a^+$ cells was characterized using: (1) visual quantitation, (2) cell distribution within the tumour, (3) correlation with other infiltrating cell types and (4) clustering patterns with associated infiltrating and/or tumour cells.

### Visual quantitation

Density was assessed by calculating the mean of 50 random fields per frozen section (field size 0.375 mm diameter, 0.11 $mm^2$, 400 × magnification) (Dunnill, 1968), enabling a representative estimation of the density and distribution of different cell types in the infiltrate to be compared with $CD1a^+$ cells.

### Distribution assessment

Location of positive cells in relation to tissue landmarks such as ducts, stroma or tumour cell nests enabled comparisons of different distribution patterns to be made between different histological grades, as well as between cells positive for different MAbs.

### Statistics

Non-parametric statistics were used for all calculations. For tests of statistical significance between groups, the Kruskal–Wallis analysis of variance and Mann–Whitney *U*-tests were used. Correlations were analysed with the Spearman rank linear correlation test.

### RESULTS

S-100 was observed to react with both putative DCs and epithelial elements in breast tissues, which therefore made specific interpretation difficult, while CD1a specifically stained a population of cells with a predominantly typical dendritic morphology, as well as occasional cells that did not appear to have dendrites (Figure 1). As a result of these findings, CD1a was used in all staining experiments to identify putative DCs.

### Density and distribution of $CD1a^+$ cells

#### *Density*

$CD1a^+$ cells were present in 84% of tumour samples ($n$ = 52), and in 22% of non-tumour samples ($n$ = 9). The density of the infiltrate was highly variable between individual tumours (range 0–46 per

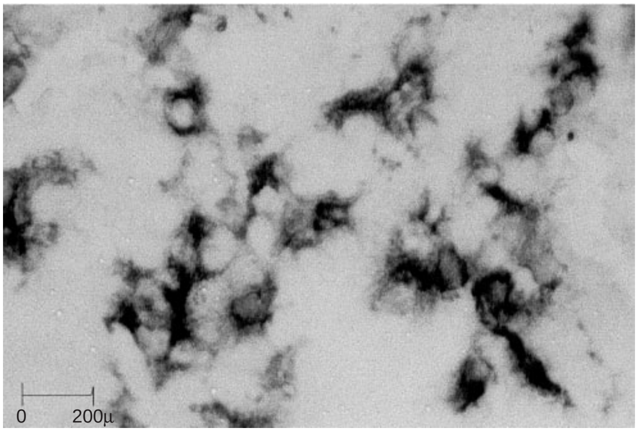

**Figure 1** High-power photomicrograph of $CD1a^+$ cells in 5 μm frozen sections of human breast tumour. The cells show a predominantly dendritic morphology. Most assume irregular shapes, although some appear to be rounded. This may represent their orientation in the plane of the tissue section (600 × magnification)

© Cancer Research Campaign 1999 

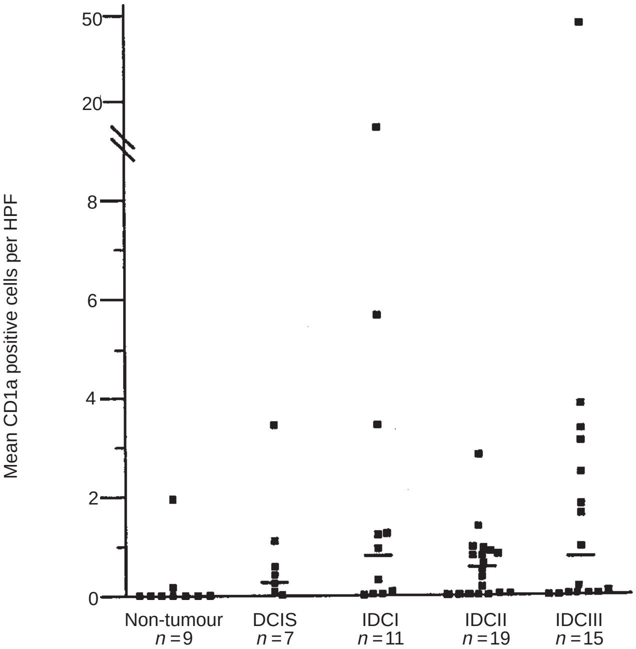

**Figure 2** The density of $CD1a^+$ cells in ductal carcinoma of the breast. There was no significant difference in density between tumour grades but, in the non-tumour tissue, the infiltrate was significantly lower or absent ($P < 0.05$). Eighty-four per cent of the tumour samples had an infiltrate of $CD1a^+$ cells compared with 22% of non-tumour samples. The results are expressed as mean cell counts per high-power field (HPF), with 50 HPFs counted per sample at 400 × magnification. Bar represents median values high-power field), but there was no correlation with tumour grade (Mann–Whitney, $P > 0.05$). $CD1a^+$ cell density was significantly higher in tumour than in non-tumour tissue (Kruskal–Wallis, $P = 0.0192$, $n = 61$). Figure 2 shows the density of $CD1a^+$ cell infiltrate by tumour grade. There was no correlation between $CD1a^+$ cell density and tumour grade.

### *Distribution*

Visual assessment identified periductal distribution of $CD1a^+$ cells in low-grade tumours, with $CD1a^+$ cells intermixed with tumour cells in higher grade tumours. In both low- and high-grade tumours, $CD1a^+$ cells appeared to make contact with tumour cells. The $CD1a^+$ tumour cell association was demonstrated with a double staining technique using anti-CD1a and anti-CAM5.2, with the result that in 90% of tumours the $CD1a^+$ cells were clustering with CAM $5.2^+$ cells. Figure 3 shows this CD1a/CAM5.2 cell association. $CD1a^+$ cells were predominantly located either periductally or within the ducts, whereas $CD3^+$ cells were predominantly located in the tumour stroma (Figure 4).

### DISCUSSION

In the normal breast tissue microenvironment, $CD1a^+$ putative DCs are present in very low numbers, if at all. This study has identified $CD1a^+$ cells with predominantly dendritic morphology within the immune cell infiltrate of 84% of the breast tumours examined. It is highly likely that these cells are DCs, with the expression of CD1a molecules indicating that these cells are actively involved in antigen recognition and capture. The identification in this study of significantly higher densities of $CD1a^+$ cells

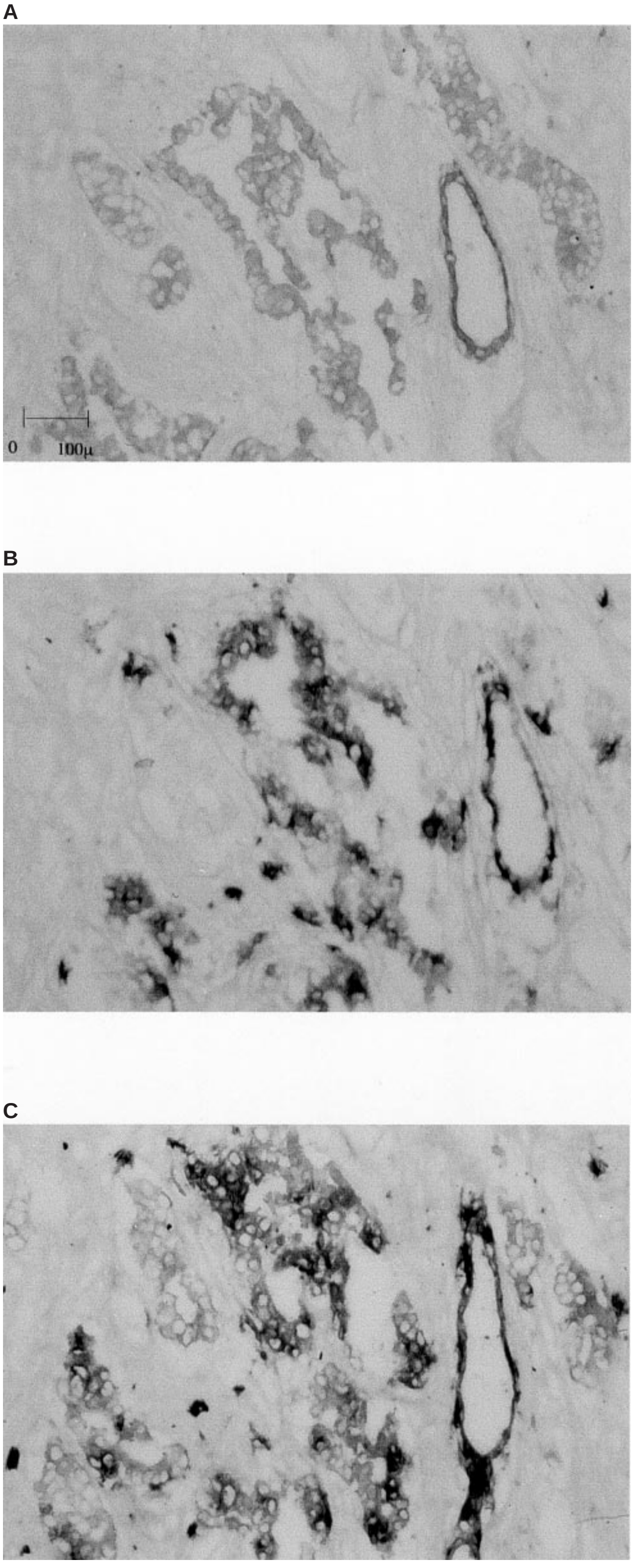

**Figure 3** (**A**) A 5 μm frozen section of a low-grade human ductal breast carcinoma. Tumour cells are identified using Cam 5.2 and diaminobenzedine/imidazole (50 × magnification). (**B**) Serial section showing darker staining $CD1a^+$ cells, visualized using diaminobenzedine/nickel chloride (50 × magnification). (**C**) Double-stained section, showing both $CD1a^+$ (darker cells) and Cam $5.2^+$ (lighter cells) in close association with each other (50 × magnification)

 © Cancer Research Campaign 1999

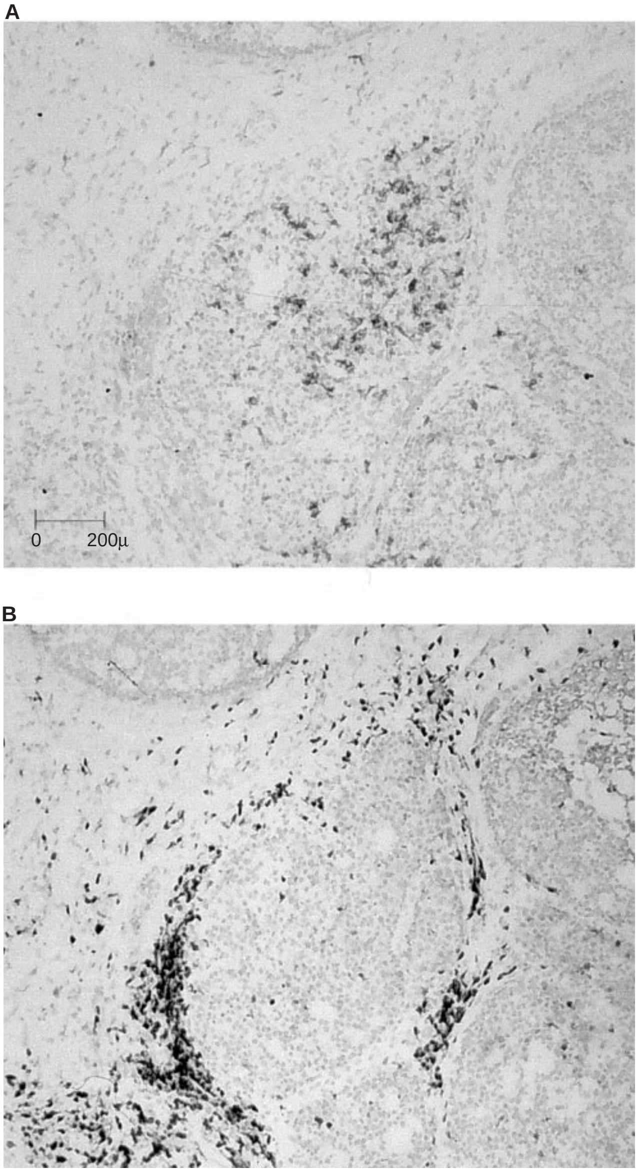

**Figure 4** (**A**) Frozen section of ductal carcinoma in situ, showing an infiltration of a transformed duct by CD1a⁺ cells. The surrounding tumour free stroma is devoid of CD1a⁺ cells (25 × magnification). (**B**) CD3⁺ cells and their distribution near the same duct. It can be clearly seen that these cells are located periductally and within the tumour-free stroma (25 × magnification)

in breast tumour tissue than in normal breast tissue indicates that in breast cancer, as in other tumours studied, CD1a⁺ cells are attracted to the tumour environment where they have the potential to participate in the immune response against the tumour. It is unclear whether DC entry is a primary or secondary event due to cytokines produced by either monocyte/macrophages, lymphocytes and/or transformed cells. Both granulocyte–macrophage colony-stimulating factor (GM-CSF) and IL-2 have been shown to induce migration of DCs into tissues (Kradin et al, 1996). IL-2R is expressed by DCs in rats and anti-IL-2R has been shown to block IL-2-induced DC migration (Kradin et al, 1996). IL-10 release from tumour cells may be important in inactivation of CD1a-positive cells (Enk et al, 1997). However, IL-10 was expressed at low levels in tumour and non-tumour tissue (unpublished).

The evidence regarding the origin of these cells is conflicting, with some studies indicating that tissue (or intratumoral) DCs and LCs may be derived from separate lineages (Steinman et al, 1997; Strunk et al, 1997). Alternatively, there is some emerging evidence from lymphoscintigraphic studies to suggest that lymph, and presumably LCs, from breast skin may drain into the breast tissue in transit to the axillary lymph nodes (Borgstein et al, 1997; Giuliano et al, 1997). This theory gains some support from the observation that LCs are CD1a positive and have been shown to lose Birbeck granules (BGs) following 24 h culture (Richters et al, 1994), a finding that indicates that the tissue microenvironment can influence the phenotype of LCs (or DCs). In addition, CD1a-positive LCs lacking BGs have been identified in the skin (Mommaas et al, 1994; Richters et al, 1994).

A close spatial association was demonstrated between CD1a⁺ cells and tumour cells using double staining with CD1a and CAM 5.2 antibodies, confirming observations by others in different tumour types (Kato et al, 1990; Kondo et al, 1990; Chaux et al, 1993). Periductal distribution of CD1a⁺ cells was noted in DCIS and grade I tumours, and in higher grade tumours CD1a⁺ cells were intermixed with tumour cells. This study did not show a correlation between CD1a⁺ cell density and tumour grade, whereas some studies of other tumour types have found negative correlations between tumour grade and DC infiltrate (Becker, 1992). Tumours that have shown such a negative correlation are predominantly tumours of surface epithelia, such as oesophageal (Becker, 1992), transitional cell (Inoue et al, 1993), cervical (Nakano et al, 1989) and gastric carcinoma (Huang et al, 1990; Tsujitani et al, 1992). These tissues are normally exposed to external antigens, and therefore contain a population of DCs as part of their normal cellular composition, which may partly explain the discrepancy in experimental outcomes.

It is also worth noting that differences in experimental conditions, which have not been consistent between studies, may have contributed to differences between results. High-sensitivity nickel-enhanced DAB detection methods used in this study are estimated to be 7–10 times more sensitive than DAB alone (Coventry et al, 1994). In addition, some studies examining DC infiltrates in tumours have used S100 (Nakano et al, 1989; Huang et al, 1990; Tsujitani et al, 1992; Inoue et al, 1993), while others have used CD1a (Rucci et al, 1991). However, it has been demonstrated that not all S100-positive cells are DCs. Barrett et al (1994) demonstrated, using a double-staining protocol, that 41–84% of the S100-positive cells in the stratum basale of the oral mucosa were also positive for melanin, indicating that these cells were probably melanocytes that could potentially be mistaken for DCs. S100 may have a cytoskeletal function, which perhaps explains its presence in cells with little in common other than dendritic morphology. Alternatively, a common embryological origin from neural crest may exist.

The inclusion of DCs in immunotherapeutic protocols is becoming feasible. Recent methods for isolation of tumour-infiltrating DCs from human breast cancers may permit functional studies of the DCs and their modulation of the anti-tumour immune response (Coventry et al, 1997). Blood DC separation and culture techniques have also advanced (Hart, 1997; Lotze et al, 1997; Schuler and Romani, 1997), and immunotherapy using DCs primed with tumour peptide antigens may offer a more specific molecular approach than conventional cancer chemotherapeutic treatments (Elsasser Beile and von Kliest, 1993). Adoptive immunotherapy

*© Cancer Research Campaign 1999*

using autologous APCs may provide a strategy for enhanced immune responses reproducing that seen in early tumour growth. Alternatively, recruitment of endogenous naive DCs using GM-CSF to promote capture of vaccinated tumour peptides (Dranoff et al, 1997) could provide an effective anti-tumour strategy.

It may also be possible to use the density of infiltrating CD1a+ cells as a prognostic indicator by relating their density to survival and recurrence rates in longitudinal follow-up studies 5 and 10 years following surgical resection. The activation status of DCs within tumours is a further topic currently under investigation.

### ACKNOWLEDGEMENTS

Funding was provided in part by the Royal Australasian College of Surgeons and the Anti-Cancer Foundation of the Universities of South Australia. Assistance in obtaining specimens for these studies from Drs P Gill, P Malycha and M Carter (surgery), E Vernon-Roberts and C Duncis (pathology) is greatly appreciated. Antibodies were kindly donated by Mr J Milios, IMVS Adelaide (S-100) and Dr L Ashman, IMVS Adelaide (isotype controls IgG1, IgG2a).

### REFERENCES

Barrett AW, Beynon A and Reid D (1994) A comparative study on tissue processing procedures for the immunohistochemical investigation of oral mucosal Langerhans cells. *Histochem J* **26**: 134–141

Becker Y (1992) Anticancer role of dendritic cells (DC) in human and experimental cancers – a review. *Anticancer Res* **12**: 511–520

Borgstein PJ, Meijer S and Pijpers R (1997) Intradermal blue dye to identify sentinel lymphnode in breast cancer. *Lancet* **349**: 1668–1669

Chaux P, Hammann A, Martin F and Martin M (1993) Surface phenotype and functions of tumour infiltrating dendritic cells: CD8 expression by a cell subpopulation. *Eur J Immunol* **23**: 2517–2525

Coventry BJ, Neoh S, Mantzioris B, Skinner JM, Bradley J and Zola H (1994) Comparison of the sensitivity of immunoperoxidase staining methods with high-sensitivity fluorescence flow cytometry–antibody quantitation on the cell surface. *J Histochem Cytochem* **42**: 1143–1149

Coventry BJ, Bradley J and Skinner JM (1995) Differences between standard and high-sensitivity immunoperoxidase staining methods in tissue sections – comparison of immunoperoxidase staining methods using computerised video image analysis. *Pathology* **27**: 221–224

Coventry BJ, Weeks S, Heckford S, Sykes P, Skinner J and Bradley J (1996) Lack of interleukin-2 (IL-2) cytokine expression despite IL-2mRNA transcription in tumour infiltrating lymphocytes in primary human breast carcinoma: selective expression of early activation markers. *J Immunol* **156**: 3486–3492

Coventry BJ, Austyn JM, Chryssidis S, Hankins D and Harris A (1997) Identification and isolation of CD1a positive putative tumour infiltrating dendritic cells in human breast cancer. In *Dendritic Cells in Fundamental and Clinical Immunology*, Vol. 3. Ricciardi-Castagnoli P. (ed) Plenum Press: NY. *Adv Exp Med Biol* **417**: 571–577

Dranoff G, Soiffer R, Lynch T, Mihm M, Jung K, Kolesar K, Liebster L, Lam P, Duda R, Mentzer S, Singer S, Tanabe K, Johnson R, Sober A, Bhan A, Clift S, Cohen L, Parry G, Rokovich J, Richards L, Drayer J, Berns A and Mulligan RC (1997) A phase I study of vaccination with autologous irradiated melanoma cells engineered to secrete human granulocyte macrophage-colony stimulating factor. *Hum Gene Ther* **8**: 111–123

Dunnill MS (1968) Quantitative methods in histology. In *Recent Advances in Clinical Pathology*, series 5. Dyke SC (ed). Churchill: London

Elsasser-Beile U and von Kliest S (1993) Cytokines as therapeutic and diagnostic agents. *Tumor Biol* **14**: 69–94

Enk AH Jonuleit H, Saloga J and Knop J (1997) Dendritic cells as mediators of tumour-induced tolerance in metastatic melanoma. *Int J Cancer* **73**: 309–316

Feradini L, Mackensen A, Genevee C, Bosq J, Duvillard P, Avril M-F and Hercend T (1993) Analysis of T-cell receptor variability in tumour infiltrating lymphocytes from a human regressive melanoma. *J Clin Invest* **91**: 1183–1190

Giuliano AE, Jones RC, Brennan M and Statman R (1997) Sentinel lymphadenectomy in breast cancer. *J Clin Oncol* **15**: 2345–2350

Hanau D, Fricker D, Bieber T, Esposito-Farese M, Bausinger H, Cazenave J, Donato L, Tongio M and de la Salle H (1994) CD1 is not affected by human peptide transporter deficiency. *Hum Immunol* **41**: 61–68

Hart DN (1997) Dendritic cells: unique lymphocyte populations which control the primary immune response. *Blood* **90**(9): 3245–3287

Head JF, Elliot R and McCoy JL (1993) Evaluation of lymphocyte immunity in breast cancer patients. *Breast Cancer Res Treat* **26**: 77–88

Huang JA, Huang HD, Peng QB, Zhu ZJ and Yu XR (1990) S100 protein positive dendritic cells and the significance of their density in gastric precancerous lesions. *Proc Chinese Acad Med Sci Peking Union Med Coll* **5**: 93–96 (abstract)

Inoue K, Furihata M, Ohtsuki Y and Fujita Y (1993) Distribution of S100 protein positive dendritic cells and expression of HLA-DR antigen in transitional cell carcinoma of the urinary bladder in relation to tumour progression and prognosis. *Virch Arc A Pathol Anat Histopathol* **422**: 351–355

Johnson JG and Jenkins J (1992) Co-stimulatory functions of antigen presenting cells. *J Invest Derm* **99**: 62S–65S

June CH, Bluestone JA, Nadler LM and Thompson CB (1994) The B7 and CD28 receptor families. *Immunol Today* **15**: 321–331

Kato H, Mizuno N, Nakagawa K, Furukawa M and Hamada T (1990) Microcystic adnexal carcinoma: a light microscopic, immunohistochemical and ultrastructural study. *J Cutaneous Pathol* **17**: 87–95

Kondo K, Mukai K, Sato Y, Matsuno Y, Shimosato Y and Monden Y (1990) An immunohistochemical study of thymic epithelial tumours III. The distribution of interdigitating reticulum cells and S100 beta positive small lymphocytes. *Am J Surg* **14**: 1139–1147

Kradin RL, Xia W, Pike M, Byers HR and Pinto C (1996) Interleukin-2 promotes the motility of dendritic cells and their accumulation in lung and skin. *Pathobiology* **64**: 180–186

Krenacs L, Tiszalvicz L, Krenacs T and Boumsell L (1993) Immunohistochemical detection of CD1a antigen in formalin fixed and paraffin embedded tissue sections with monoclonal antibody 010. *J Path* **171**: 99–104

Lotze MT, Shurin M, Davis I, Amoscato A and Storkus W (1997) Dendritic cell based therapy of cancer. In *Dendritic Cells in Fundamental and Clinical Immunology*, Vol 3. Ricciardi-Castagnoli P (ed). Plenum Press: NY. *Adv Exp Med Biol* **417**: 551–569

Makin CA, Bobrow LG and Bodmer WF (1984) Monoclonal antibody to cytokeratin for use in routine histopathology. *J Clin Path* **37**(9): 975–983

Mommaas M, Mulder A, Vermeer B and Koning F (1994) Functional human epidermal Langerhans cells that lack Birbeck Granules. *J Invest Dermatol* **103**: 807–810

Moulon C, Peguet-Navarro J and Schmitt D (1991) A potential role for CD1a molecules on human epidermal Langerhans cells in allogeneic T-cell activation. *J Invest Derm* **97**: 524–528

Nakano T, Oka K, Arai T, Morita S and Tsunemoto H (1989) Prognostic significance of Langerhans cell infiltration in radiation therapy for squamous cell carcinoma of the uterine cervix. *Arch Pathol Lab Med* **113**: 507–511

Osterhoff B, Rappersberger K, Wang B, Koszik F, Ochiai K, Kinet JP and Stingl G (1994) Immunomorphologic characterization of Fc epsilon RI bearing cells within the human dermis. *J Invest Derm* **102**: 315–320

Porcelli S, Brenner M, Greenstein J, Balk S, Terhorst C and Bleicher P (1989) Recognition of cluster of differentiation 1 antigens by human CD4–CD8 cytolytic T-lymphocytes. *Nature* **341**(6241): 447–450

Richters CD, Hoekstra M, van Baare J, Du Pont J, Hoefsmit E and Kamperdijk E (1994) Isolation and characterization of migratory human skin dendritic cells. *Clin Exp Immunol* **98**: 330–336

Rucci L, Bani D, Gallo O, Arbi Riccardi R, Borghi-Cirri MB and Fini-Storchi O (1991) Interdigitating cells in the peritumoral infiltrate of laryngeal carcinomas: an immunocytochemical and ultrastructural study. *J Otorhinolaryngol Relat Spec* **53**: 349–356

Schuler G and Romani N (1997) Generation of mature dendritic cells from human blood. In *Dendritic Cells in Fundamental and Clinical Immunology Vol 3* P. Ricciardi-Castagnoli (ed) Plenum Press NY. *Adv Exptl Med Biol* **417**: 7–14

Steinman RM, Pack M and Inaba K (1997) Dendritic cell development and maturation. In *Dendritic Cells in Fundamental and Clinical Immunology*, Vol 3. Ricciardi-Castagnoli P. (ed) Plenum Press NY. *Adv Exptl Med Biol* **417**: 1–6

Strunk D, Egger C, Leitner G, Hanau D and Stingl G (1997) A skin homing molecule defines the Langerhans cell progenitor in human peripheral blood. *J Exp Med* **185**(6): 1131–1136

Tsujitani S, Kakeji Y, Watanabe A, Kohnoe S, Maehara Y and Sugimaghi K (1992) Infiltration of S100 protein positive dendritic cells and peritoneal recurrence in advanced gastric cancer. *Int Surg* **77**: 238–241

Williams L, Egner W and Hart DJ (1994) Isolation and function of human dendritic cells. *Int Rev Cytol* **153**: 41–103

Yu CY and Milstein C (1989) A physical map linking the 5 CD1 human thymocyte differentiation antigen genes. *EMBO J* **8**: 3727–3732

 © Cancer Research Campaign 1999
